# Photodynamic Therapy Combined with Bcl-2/Bcl-xL Inhibition Increases the Noxa/Mcl-1 Ratio Independent of Usp9X and Synergistically Enhances Apoptosis in Glioblastoma

**DOI:** 10.3390/cancers13164123

**Published:** 2021-08-17

**Authors:** Carolin Golla, Mayas Bilal, Annika Dwucet, Nicolas Bader, Jenson Anthonymuthu, Tim Heiland, Maximilian Pruss, Mike-Andrew Westhoff, Markus David Siegelin, Felix Capanni, Christian Rainer Wirtz, Richard Eric Kast, Marc-Eric Halatsch, Georg Karpel-Massler

**Affiliations:** 1 Department of Neurological Surgery, Ulm University Medical Center, 89081 Ulm, Germany; carolin.golla@uni-ulm.de (C.G.); mayasbilal@hotmail.com (M.B.); annika.dwucet@uniklinik-ulm.de (A.D.); jenson.anthonymuthu@uni-ulm.de (J.A.); tim.heiland@outlook.de (T.H.); Maximilian.Pruss@med.uni-duesseldorf.de (M.P.); rainer.wirtz@uniklinik-ulm.de (C.R.W.); marc-eric.halatsch@ksw.ch (M.-E.H.); 2 Department of Mechatronics and Medical Engineering, Ulm University of Applied Sciences, 89081 Ulm, Germany; nicolas.bader@thu.de (N.B.); felix.capanni@thu.de (F.C.); 3 Department of Pediatric and Adolescent Medicine, Ulm University Medical Center, 89075 Ulm, Germany; andrew.westhoff@uniklinik-ulm.de; 4 Department of Pathology and Cell Biology, Columbia University Irving Medical Center, New York, NY 10032, USA; ms4169@cumc.columbia.edu; 5 IIAIG Study Center, Burlington, VT 05408, USA; richarderic.kast@gmail.com

**Keywords:** glioblastoma, 5-aminolevulinic acid, photodynamic therapy, ABT-263, navitoclax, Bcl-xL

## Abstract

**Simple Summary:**

Glioblastoma represents one of the most common malignant brain tumors in adults and is associated with a poor clinical outcome despite current therapeutic measures. Therefore, novel strategies for the treatment of this disease are urgently needed. In this work, we examined the antineoplastic effects of a combined treatment with photodynamic therapy and ABT-263 on different glioblastoma cells. Photodynamic therapy uses the selective uptake of a photosensitive molecule followed by activation by light of a specific wavelength to kill cancer cells. ABT-263 is a small molecule inhibitor that targets cancer cells by facilitating programmed cell death. This novel combinatorial therapeutic strategy synergistically killed glioblastoma cells. These results indicate that a combination of the two treatment modalities may be of benefit for the treatment of glioblastoma supporting further studies.

**Abstract:**

The purpose of this study was to assess in vitro whether the biological effects of 5-aminolevulinic acid (5-ALA)-based photodynamic therapy are enhanced by inhibition of the anti-apoptotic Bcl-2 family proteins Bcl-2 and Bcl-xL in different glioblastoma models. Pre-clinical testing of a microcontroller-based device emitting light of 405 nm wavelength in combination with exposure to 5-ALA (PDT) and the Bcl-2/Bcl-xL inhibitor ABT-263 (navitoclax) was performed in human established and primary cultured glioblastoma cells as well as glioma stem-like cells. We applied cell count analyses to assess cellular proliferation and Annexin V/PI staining to examine pro-apoptotic effects. Western blot analyses and specific knockdown experiments using siRNA were used to examine molecular mechanisms of action. Bcl-2/Bcl-xL inhibition synergistically enhanced apoptosis in combination with PDT. This effect was caspase-dependent. On the molecular level, PDT caused an increased Noxa/Mcl-1 ratio, which was even more pronounced when combined with ABT-263 in a Usp9X-independent manner. Our data showed that Bcl-2/Bcl-xL inhibition increases the response of glioblastoma cells toward photodynamic therapy. This effect can be partly attributed to cytotoxicity and is likely related to a pro-apoptotic shift because of an increased Noxa/Mcl-1 ratio. The results of this study warrant further investigation.

## 1. Introduction

Glioblastoma represents a cerebral malignancy that is very difficult to treat. In general, patients diagnosed with a glioblastoma face a median overall survival of less than two years [1,2]. This grim situation persists despite vast efforts in refining therapeutic measures such as maximal safe resection, the EORTC scheme, or alternating electrical fields [1,2]. Unfortunately, so far, the great progress that has been made in characterizing the disease on the molecular level has not translated into the desired therapeutic success. This situation can be attributed at least in part to a marked intra-tumoral heterogeneity and, in consequence, a clonal selection following therapy-induced micro-environmental changes [3]. Therefore, novel strategies need to be conceived to treat this disease more efficiently.

5-aminolevulinic acid (5-ALA) represents a precursor of the fluorescent metabolite protoporphyrin IX (PpIX) in the biosynthesis of heme. Following the administration of 5-ALA, PpIX was shown to frequently accumulate in the cells of malignant gliomas [4]. Consequently, 5-ALA is widely used for fluorescence-guided surgical removal of malignant gliomas to increase the extent of resection. Another prominent feature of 5-ALA-induced PpIX is represented by its phototoxic activity. 5-ALA-based photodynamic therapy was shown to induce cell death through various modes of action, including cell death with features of autophagy or apoptosis, in multiple pre-clinical studies of different cancers [5,6]. In the hospital setting, 5-ALA-based PDT has been applied to glioblastoma patients intra-operatively immediately following tumor resection using a balloon light diffuser [7,8]. The results from this trial (NCT03048240) are pending. Another strategy that has been clinically applied is the stereotactic placement of cylindrical diffusers within tumors (interstitial PDT), avoiding tumor resection. A preliminary study including 15 patients with a newly diagnosed glioblastoma receiving interstitial PDT reported promising results. Patients that underwent interstitial PDT had in median an overall survival of 16 months versus 10.2 months in patients receiving “complete resection” only [9]. A randomized controlled trial (NOA11; NCT04469699) is currently investigating this approach in glioblastoma patients to gain further insight into whether this therapeutic measure provides a significant benefit [10].

The intrinsic apoptotic pathway is orchestrated by a sophisticated interaction of pro- and anti-apoptotic Bcl-2 family proteins [11]. While under normal circumstances this network is tightly balanced, in malignancies an anti-apoptotic shift is frequently perceived as the result of upregulation of, for instance, Bcl-2, Mcl-1, or Bcl-xL. BH-3 mimetics like navitoclax (ABT-263) were developed as inhibitors of Bcl-2 and Bcl-xL, reinstating a pro-apoptotic phenotype [12]. This BH-3 mimetic-induced change of the apoptotic phenotype is caused by an impairment of the sequestration of BAX and/or BAK and by a displacement of pro-apoptotic molecules (i.e., Noxa or BAD) from anti-apoptotic proteins of this family (i.e., Mcl-1 or Bcl-2) [13]. From a translational perspective, navitoclax has been already clinically studied as a therapeutic for various human malignancies in multiple trials [14,15,16,17,18].

In this study, PDT synergistically enhanced the pro-apoptotic activity of the inhibitor of Bcl-2 and Bcl-xL, ABT-263. On the molecular level, this effect is accompanied by an increased Noxa/Mcl-1 ratio as a result of a transcriptionally mediated decrease in Mcl-1 and enhanced protein stability of Noxa. Overall, additional treatment with BH-3 mimetics such as ABT-263 may increase the therapeutic efficacy of PDT and seems worthy of consideration in further studies.

## 2. Materials and Methods

### 2.1. Reagents

ABT-263 was obtained from Selleckchem (Houston, TX, USA). ABT-263 was dissolved with dimethylsulfoxide at a 10 mM concentration for stock. The stocks were refrigerated at −20 °C. The final concentrations of dimethylsulfoxide were less than 0.1% (*v*/*v*).

### 2.2. Cell Cultures and Growth Conditions

U251 glioblastoma cells were obtained from Sigma Aldrich 01/2017 (St. Louis, MO, USA). The primary cultures ULM-GBM-PC128, ULM-GBM-PC38, as well as ULM-GBM-PC40, and the glioblastoma cells ULM-GBM-SC128, ULM-GBM-SC38, and ULM-GBM-SC40 with stem-like features were cultivated from tumor tissue obtained from patients that were operated on at our hospital [19,20,21,22,23]. U251 and the primary cultures were maintained in Dulbecco’s modified Eagle’s medium (DMEM; GIBCO, Invitrogen, Paisley, UK) containing 10% fetal bovine serum (FBS), 100 IU/mL penicillin, 100 µg/mL streptomycin, 4 mM glutamine, 1 mM sodium pyruvate (GIBCO, Invitrogen, Grand Island, NY, USA) as described before [24,25,26]. The stem-like phenotype of the glioblastoma stem-like cells was preserved by maintaining cells as sphere cultures in DMEM/F-12 (HAM) medium (Gibco, Life Technologies, Darmstadt, Germany) supplemented with human recombinant epidermal growth factor (Biomol GmbH, Hamburg, Germany), human recombinant basic fibroblast growth factor (Miltenyi Biotec GmbH, Bergisch Gladbach, Germany) and serum-free neuron culture supplement B27 (Gibco, Life Technologies). All cells were incubated at 37 °C in a water-saturated atmosphere containing 5% CO_2_. The procedures were approved by the ethics committee of the University of Ulm (No.162/10) and consent was granted by the patients or next of kin.

### 2.3. Photodynamic Therapy

An implantable microcontroller-based LED device was used for the emission of light at the wavelength of 405 nm, a radiation power (Φ_e_) of 6.8 mW and an irradiance (E_e_) of 4.8 mJ (cm^2^ s)^−1^. A miniaturized version of this device was successfully tested intracerebrally in a safety and tolerability study in a porcine model previously (http://www.biomechanik-ulm.de/) (accessed on 10 July 2021). For the photodynamic therapy, treatment with 25 µg/mL 5-aminolevulinic acid was performed for 4 h followed by exposure to light at the wavelength of 405 nm for indicated durations of time.

### 2.4. Measurement of Apoptosis

Staining with Annexin V and Propidium iodide prior to flow cytometry was used to determine apoptosis [27,28]. Four × 10^4^ cells were seeded on 12-well plates. Following the specific treatments, the supernatants were collected. Adherent cells were detached with Trypsin/EDTA (Biochrom AG, Berlin, Germany) and pooled with the respective supernatants. The cells were centrifuged and washed twice with ice-cold Annexin V binding buffer prior to being resuspended in binding buffer. Annexin-V-FLUOS (Roche Diagnostics, Indianapolis, IN, USA) was added prior to incubation for 15 min at room temperature. Before each measurement, Propidium iodide was added to a final concentration of 2.5 μM. Flow cytometry was performed with the FACSCantoTM II (BD Biosciences, NJ, USA) recording 10,000 events. Further analysis was done with FlowJo version 8.7.1 (Tree Star, Ashland, OR, USA).

### 2.5. Cell Count Analyses

Cell count analyses were performed as described before [29]. One × 10^4^ cells were plated per well. After distinct periods of time, an aliquot of cells growing in suspension was collected and cellular concentrations were determined with a cell counter. For adherent cells, the medium was aspirated, followed by enzymatical detachment (Trypsin/EDTA, Biochrom AG, Berlin, Germany) and further analysis as performed for cells growing in suspension.

### 2.6. Measurement of Intracellular Reactive Oxygen Species (ROS)

Staining with 2′,7′-Dichlorodihydrofluorescein diacetate (Sigma Aldrich, St. Louis, MO, USA) followed by flow cytometric analysis were performed to quantify ROS formation. Four × 10^4^ cells were seeded on 12-well plates and subjected to the specific treatments. Following defined periods of time, 2′,7′-Dichlorodihydrofluorescein diacetate was added at a concentration of 10 µM prior to incubation for 10 min at 37 °C, avoiding exposure to light. Then cells were washed twice with PBS and enzymatically detached using Trypsin/EDTA. Afterwards, the cells were collected, centrifuged, and washed once with PBS prior to resuspension in PBS and flow cytometric analysis (FACSCantoTM II). For each measurement 10,000 events were recorded. Further analysis was done with FlowJo version 8.7.1.

### 2.7. Western Blot Analysis

Expression of specific cellular proteins was determined by Western blot as previously described [30,31]. Briefly, for cell lysis a buffer was used containing 150 mM NaCl, 1% Triton X-100, 10% glycerol, 30 mM Tris–HCl (pH 7.4), 200 mM phenylmethylsulfonyl fluoride, 2 mM dithiothreitol, 1 mM 3-glycerophosphate, 1 mM Na_2_VO_3_, 50 mM NaF and the Complete Protease Inhibitor Cocktail (Roche Diagnostics GmbH). The cell lysates were transferred to Eppendorf tubes and denaturation was performed at 70 °C for 10 min prior to storage at −20 °C. Fifty micrograms per sample were separated on SDS-PAGE (6% for Usp9X and 12% for all other proteins) and transferred to a nitrocellulose membrane (Amersham) before incubation with specific antibodies. The Pierce^TM^ enhanced chemiluminescence Western blotting substrate was used for detection of target proteins and documentation with the Fusion FX chemiluminescence imaging system (Vilber Lourmat, Eberhardzell, Germany). The following primary antibodies were used: Bcl-2 (#551098, 1:1000; BD Pharmingen, San Diego, CA, USA), Mcl-1 (#5453S, clone: D35A5, 1:1000; Cell Signaling Technology, Danvers, MA, USA), Bcl-xL (#2764, clone 54H6, 1:500; Cell Signaling Technology), Noxa (#556585, clone 114C307.1, 1:1000; Enzo Life Sciences, Farmingdale, NY, USA), Usp9X (#5751S, 1:1000; Cell Signaling Technology, Danvers, MA, USA), caspase-3 (#9662, 1:1000; Cell Signaling Technology), caspase-9 (#9508T, clone C9, 1:1000; Cell Signaling Technology), Actin (clone AC15, 1:2000; Sigma Aldrich, St. Louis, MO, USA). Secondary antibodies were obtained from Cell Signaling Technology (#7074S, #7076S).

### 2.8. Transfections of siRNAs

siRNAs were transfected as previously described [32] and according to the manufacturers’ instructions using Viromer^®^ BLUE (Lipocalyx, Halle, Germany) as transfection reagent. Four × 10^4^ cells were seeded on 12-well plates. After 24 h, the medium was aspirated, and the cells were incubated with the formed complexes of Viromer^®^ BLUE (Lipocalyx, Halle, Germany) and the respective siRNA in DMEM with 1.5% FBS until flow cytometry and Western blot analysis. siRNA-targeting Bcl-xL was purchased from CST (Bcl-xL-siRNA I, SignalSilence^®^, Danvers, MA, USA). NOXA-siRNA (Silencer^®^ Select, s10709) was purchased from Ambion (Life Technologies Corporation, Carlsbad, CA, USA). Non-targeting siRNA was obtained from Dharmacon (D-001810-03-05, ON-TARGETplus, Lafayette, CO, USA).

### 2.9. cDNA Synthesis and Real-Time Quantitative PCR

The Maxwell^®^ 16 LEV simplyRNA kit (Promega) was used according to the manufacturer’s instruction to extract total RNA. The Biozym cDNA Synthesis Kit (Biozym Scientific GmbH, Hess Oldendorf, Germany) was used for reverse transcription of 0.5 µg of RNA per sample. Real-time quantitative PCR was performed on the StepOnePlus^TM^ Real-Time PCR System (Applied Biosystems Inc., Waltham, MA, USA) using the SsoAdvanced^TM^ Universal SYBR^®^ Green Supermix (Bio-Rad Laboratories GmbH, Feldkirchen, Germany). The following conditions were used: 30 s at 95 °C followed by 40 cycles of 10 s at 95 °C and 20 s at 60 °C. Fold changes were calculated relative to 18S as endogenous control. The primer sequences are presented in Table 1.

### 2.10. Statistical Analysis

One-way ANOVA followed by Newman–Keuls post hoc analysis were used to test for statistical significance (GraphPad Prism version 5.04, La Jolla, CA, USA). Statistical significance was assumed for *p*-values < 0.05. We performed BLISS analysis to further characterize the interaction between the different treatment modalities [19,33]. The expected total response was calculated as fractional response to drug A (Fa) + fractional response to drug B (Fb) − Fa × Fb. A ratio of the actual total response and the expected total response of 0.9 to 1.1 was considered as additive, a ratio <0.9 as antagonistic, and a ratio >1.1 as synergistic.

## 3. Results

### 3.1. PDT with a Microcontroller-Based Device Has a Dose-Dependent Pro-Apoptotic Effect on Glioblastoma Cells

**Figure 1:**
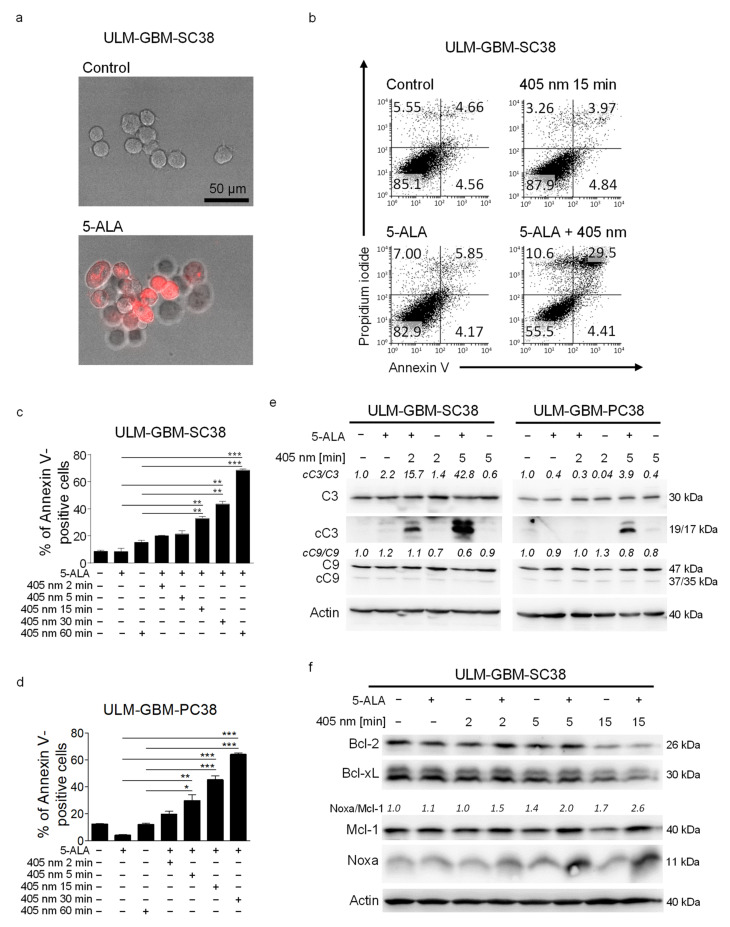
PDT induces apoptosis and causes an increased Noxa/Mcl-1 ratio. (**a**) Representative fluorescence microphotographs of ULM-GBM-SC38 cells treated with 25 µg/mL 5-ALA for 4 h prior to exposure to light at a wavelength of 405 nm, magnification 20× *g*. (**b**) Flow plots of ULM-GBM-SC38 cells subjected to solvent, 25 µg/mL 5-ALA, light with a wavelength of 405 nm or both for 24 h. Staining with Annexin V/Propidium iodide and flow cytometry was performed. (**c**,**d**) Quantitative representation of ULM-GBM-SC38 and ULM-GBM-PC38 cells subjected to the same treatment as outlined in b. Columns, mean; bars, SD; *n* = 3. * *p* < 0.05, ** *p* < 0.01, *** *p* < 0.005. (**e**) ULM-GBM-SC38 and ULM-GBM-PC38 cells were treated for 6 h as indicated. Cell extracts were collected and analyzed by Western blot for caspase 3 (C3), cleaved caspase 3 (cC3), caspase 9 (C9), and cleaved caspase 9 (cC9) expression. Actin served as loading control. Image J (NIH, Bethesda, MD; http://imagej.nih.gov/ij (accessed on 10 July 2021).) was used for densitometric analysis. (**f**) ULM-GBM-SC38 cells were treated for 24 h as indicated. Cell extracts were collected and analyzed by Western blot for Bcl-2, Bcl-xL, Mcl-1 and NOXA expression. Protein loading was controlled by Western blot for Actin. Densitometric analysis and normalization was performed as described for (**e**). The uncropped western blots are in Figure S2.

Photodynamic therapy using various photosensitizing compounds has already long been observed by others to act cytocidal on a variety of malignant cells. In our study, we used 5-ALA as a photosensitizer because it is widely used in patients with a glioblastoma for fluorescence-guided tumor resection. First, we verified that cells exposed to 5-ALA showed uptake of 5-ALA in our setting. As shown in Figure 1a, the majority of ULM-GBM-SC38 cells that were exposed to 5-ALA showed a strong fluorescent signal when stimulated with light at a wavelength of 405 nm. Next, we examined whether exposure to 405 nm light emitted by a microcontroller-based LED device would lead to enhanced apoptosis in cells that were pre-treated with 5-ALA. Photodynamic therapy significantly increased the fraction of apoptotic (Annexin V-positive) ULM-GBM-SC38 cells (Figure 1b). Moreover, the pro-apoptotic effect of PDT increased dose-dependently in primary cultured ULM-GBM-PC38 cells and glioma stem-like ULM-GBM-SC38 cells (Figure 1c,d). In line with this observation, Western blot analyses showed enhanced cleavage of caspase 3 following PDT when compared to either treatment with 5-ALA or treatment with light at 405 nm wavelength alone (Figure 1e).

### 3.2. PDT Induces an Increased Noxa/Mcl-1 Ratio

Programmed cell death can be activated through the mitochondrial pathway. Therefore, we next addressed the question of whether regulatory proteins of the mitochondrial pathway are affected by PDT. As shown in Figure 1f, PDT did not significantly alter the protein levels of Bcl-2 and Bcl-xL, anti-apoptotic members of the Bcl-2 family, when compared to exposure to 405 nm light at the respective durations. In contrast, protein expression of Mcl-1 was increased in PDT-treated cells. The longer the cells were exposed to 405 nm light, the stronger was this finding. The most prominent feature we observed in ULM-GBM-SC38 cells subjected to PDT was upregulation of Noxa and a markedly enhanced Noxa/Mcl-1 ratio, which increased with the length of exposure to 405 nm light (Figure 1f).

### 3.3. Treatment with PDT and ABT-263 Synergistically Induces Apoptosis

**Figure 2:**
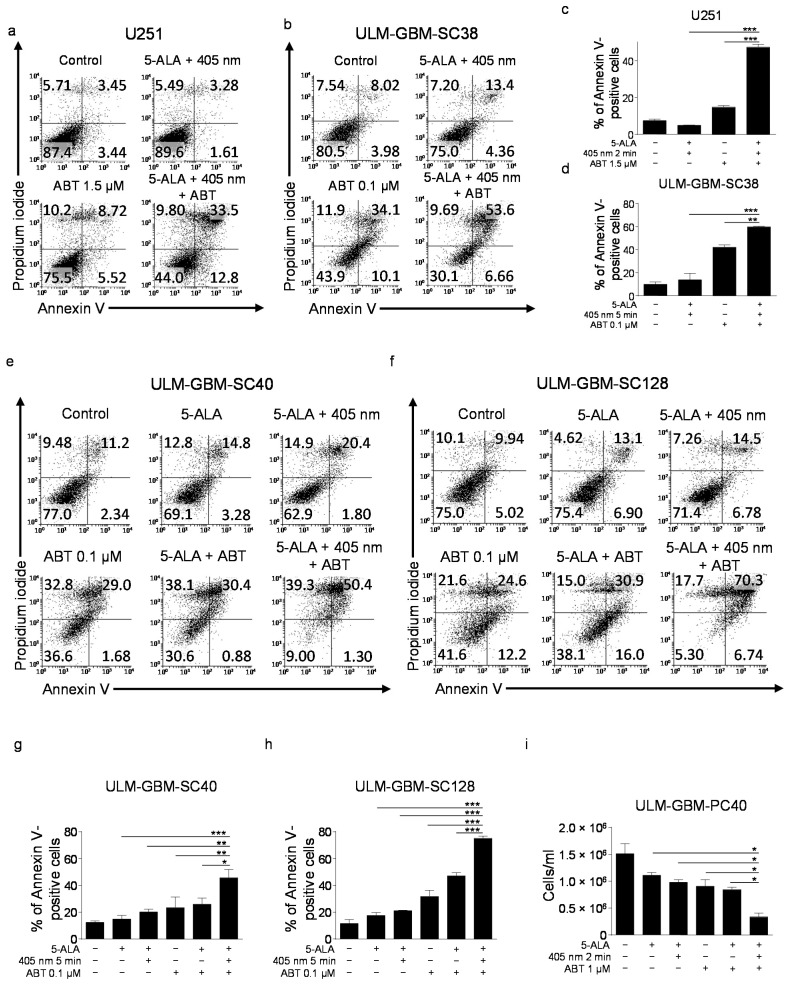
Combined treatment with PDT and ABT-263 has enhanced pro-apoptotic activity. (**a**,**b**) U251 (**a**) and ULM-GBM-SC38 (**b**) cells were subjected to treatment as outlined for 24 h. U251 cells were irradiated for 2 min and ULM-GBM-SC38 for 5 min followed by Annexin V/Propidium iodide staining and flow cytometry. (**c**,**d**) Column graphs of U251 (**c**) and ULM-GBM-SC38 (**d**) cells subjected to the same treatment as outlined in (**a**,**b**). Columns, mean of three technical replicates; bars, SD. Data are representative for three biologically separate experiments. ** *p* < 0.01, *** *p* < 0.005. (**e**,**f**) Representative flow plots of ULM-GBM-SC40 (**e**) and ULM-GBM-SC128 (**f**) cells subjected to indicated treatments for 24 h. The duration of irradiation was 5 min for both cells. Annexin V/Propidium iodide staining was done prior to flow cytometry. (**g**,**h**) Column graphs of ULM-GBM-SC40 and ULM-GBM-SC128 cells subjected to the same treatment as outlined in (**e**,**f**). Columns, mean of three technical replicates; bars, SD. Data are representative for three biologically separate experiments. * *p* < 0.05, ** *p* < 0.01, *** *p* < 0.005. (**i**) ULM-GBM-PC40 cells were treated as indicated for 24 h followed by cell count analyses. Columns, mean of three technical replicates; bars, SD. Data are representative for three biologically separate experiments. * *p* < 0.05.

We next addressed the question of whether PDT can sensitize glioblastoma cells to ABT-263. As shown in Figure 2, treatment with PDT and ABT-263 led to a statistically significant increase in apoptotic (Annexin V-positive) U251 established glioblastoma cells (Figure 2a,c) and multiple different glioblastoma cells with stem-like features (Figure 2b,d–h). Furthermore, Bliss analysis revealed that this effect was synergistic across all cells tested (Table 2). Notably, the strongest synergistic effect of the combination was found in U251.

In line with our flow cytometric analyses, cell count analyses showed a significant reduction of the cellular concentration of ULM-GBM-PC40 cells that were subjected to ABT-263 and PDT treatment combined (Figure 2i).

### 3.4. The Enhanced Apoptotic Effect of PDT Combined with ABT-263 Is Caspase-Dependent

**Figure 3:**
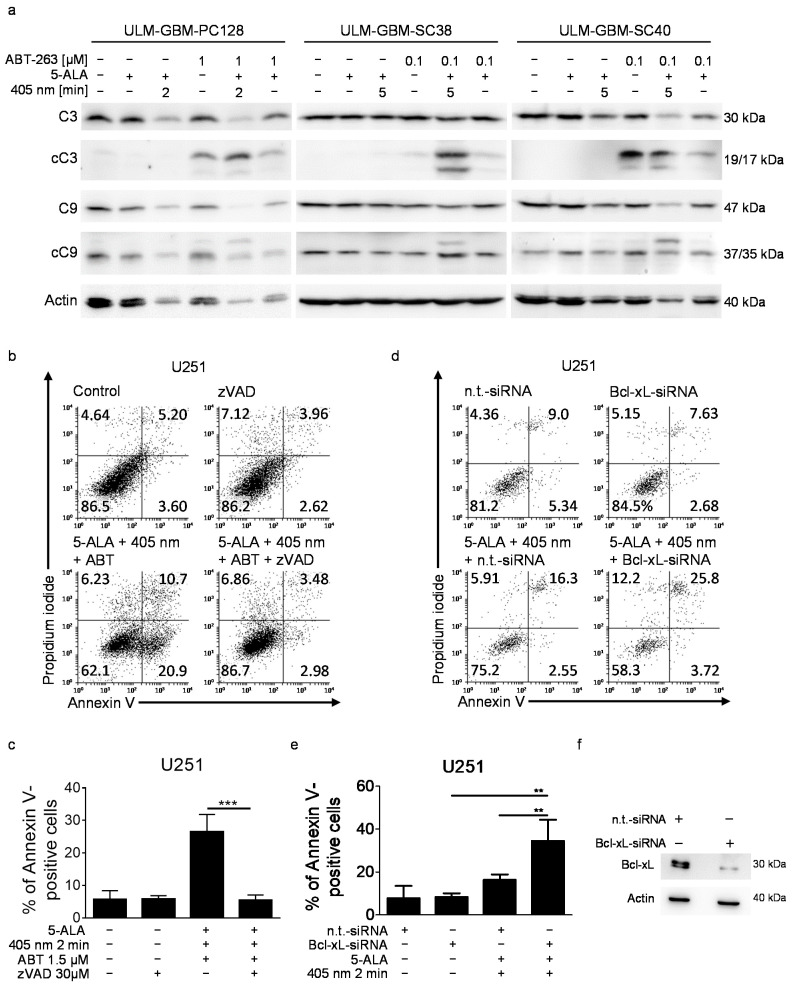
PDT combined with ABT-263 treatment induces activation of caspases and enhanced apoptosis. (**a**) ULM-GBM-PC128, ULM-GBM-SC38, and ULM-GBM-SC40 cells were subjected to treatment for 6 h as outlined. Cellular extracts were collected, and Western blot analysis was performed for caspase 3 (C3), cleaved caspase 3 (cC3), caspase 9 (C9), and cleaved caspase 9 (cC9) expression. Actin expression was used to control for loading. (**b**) Flow plots of U251 cells treated for 24 h with solvent, 1.5 µM ABT-263 plus PDT with or without 30 µM zVAD.fmk that were stained with Annexin V/Propidium iodide and analyzed by flow cytometry. (**c**) Column graphs of U251 cells that were subjected to the same treatment as outlined in (**b**). Columns, mean; bars, SD. *n* = 3. *** *p* < 0.005. (**d**) Flow plots of U251 cells that were incubated with non-targeting (n.t.)-siRNA or Bcl-xL-siRNA and treated with 1 µM ABT-263 plus PDT or solvent for 24 h. Staining with Annexin V/Propidium iodide was performed followed by flow cytometry. (**e**) Quantitative representation of U251 cells subjected to the same treatment as outlined in (**d**). Columns, mean. Bars, SD. *n* = 3. ** *p* < 0.01. (**f**) A sufficient silencing of Bcl-xL was confirmed by Western blot analysis. The uncropped western blots are in Figure S2.

We next examined whether the increased induction of apoptosis that followed the combination therapy was caspase-dependent. While treatment with ABT-263—with or without 5-ALA—led to some cleavage of caspase 3, simultaneous treatment with PDT and ABT-263 markedly raised caspase 9 as well as caspase 3 cleavage (Figure 3a). Notably, PDT alone had only a slight effect on the cleavage of the two caspases under these conditions when compared to the combination therapy. To examine whether the increased pro-apoptotic action of PDT plus ABT-263 was dependent on caspase activation, we subjected U251 glioblastoma cells to the combination treatment with or without pan-caspase inhibition by zVAD.fmk (Figure 3b,c). As shown before, combined treatment with PDT and ABT-263 significantly raised the apoptotic (Annexin V-positive) fraction of cells. However, additional treatment with zVAD.fmk completely suppressed this response.

### 3.5. Combined Treatment with PDT and ABT-263 Does Not Enhance the Formation of ROS

The formation of reactive oxygen species (ROS) is considered to represent an important part of the mechanism that underlies the antineoplastic activity of PDT [8]. Therefore, we examined how treatment with PDT, ABT-263, and the combination affected the formation of ROS. As shown in Figure S1, ABT-263-treated cells showed no increase in the generation of ROS. However, as early as 6 h after treatment with PDT alone, a significant increase in ROS was observed. Notably, cells treated with the combination of PDT and ABT-263 showed a significantly reduced formation of ROS when compared to cells treated only with PDT.

### 3.6. Specific Downregulation of Bcl-xL in Combination with PDT Leads to Enhanced Apoptosis

Next, we examined if targeting of Bcl-xL in combination with PDT was sufficient to induce an increased apoptotic response. Specific siRNA was used to suppress the expression of Bcl-xL in U251 cells before treatment with solvent or PDT (Figure 3d–f). Selective downregulation of Bcl-xL in combination with PDT significantly increased the fraction of apoptotic (Annexin V-positive) cells (Figure 3d,e).

### 3.7. Combined Treatment with PDT and ABT-263 Further Raises the Noxa/Mcl-1 Ratio

**Figure 4:**
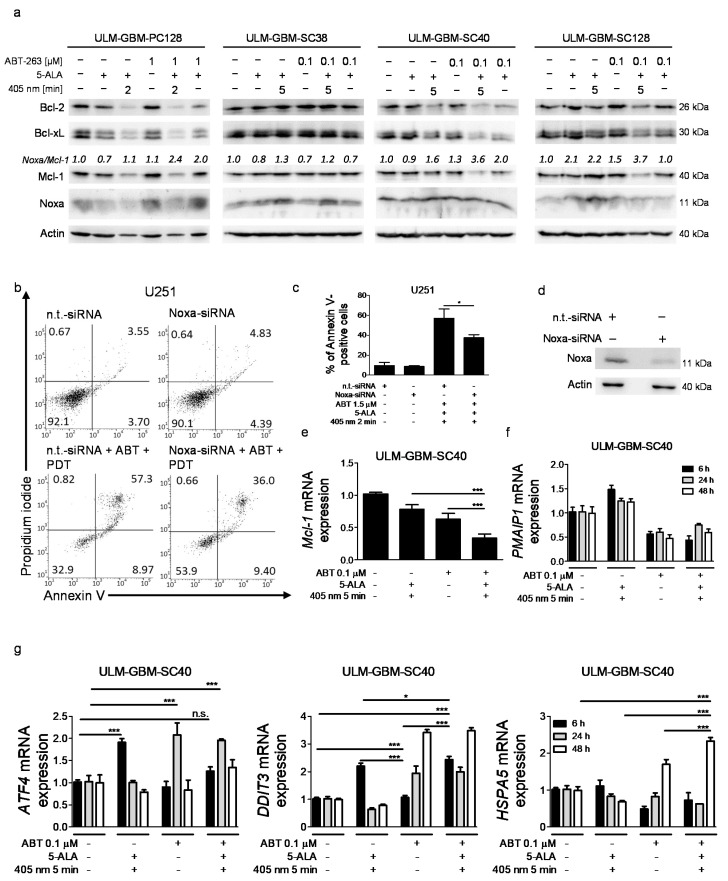
PDT combined with ABT-263 induces a further increase in the Noxa/Mcl-1 ratio. (**a**) Glioblastoma cells received different treatments as indicated for 24 h. Cell lysates were collected prior to analysis of Bcl-2, Bcl-xL, Mcl-1, and Noxa protein expression by Western blot. Loading was controlled by Actin. Image J (NIH, Bethesda, MD; http://imagej.nih.gov/ij (accessed on 10th of July 2021)) was used for densitometry. Normalization to control and the corresponding Actin signal was done. (**b**) U251 cells were incubated with non-targeting (n.t.)-siRNA or Noxa-siRNA and treated with PDT plus 1 µM ABT-263 or solvent for 24 h. Flow plots of cells stained with Annexin V/Propidium iodide are shown. (**c**) Column graphs of U251 cells subjected to the same treatment as outlined in (**b**). Columns, mean. Bars, SD. *n* = 2. * *p* < 0.05. (**d**) A sufficient knockdown of NOXA was confirmed by Western blot. (**e**) ULM-GBM-SC40 cells received the indicated treatments for 24 h. Mcl-1 mRNA expression was determined by rt-PCR. Columns, mean of three technical replicates; bars, SD. *** *p* < 0.005. (**f**) ULM-GBM-SC40 cells were treated for indicated durations of time. rt-PCR was performed for *PMAIP1*. Columns, mean of three technical replicates; bars, SD. (**g**) ULM-GBM-SC40 cells were treated for indicated durations of time. rt-PCR was performed for *ATF4*, *DDIT3* and *HSPA5*. Columns, mean of three technical replicates; bars, SD. * *p* < 0.05, *** *p* < 0.005, n.s. = non-significant. The uncropped western blots are in Figure S2.

We initially observed that PDT alone affected the Noxa/Mcl-1 ratio, shifting the cellular homeostasis toward a pro-apoptotic phenotype. Therefore, we decided to examine how the combination therapy affected the expression of anti- and pro-apoptotic proteins of the Bcl-2 family, such as Mcl-1 and Noxa. While the expression of Bcl-2 and Bcl-xL was reduced by the combination treatment in ULM-GBM-PC128 and ULM-GBM-SC40 cells, the most consistent finding was an increased Noxa/Mcl-1 ratio (Figure 4a). Notably, this increase in the Noxa/Mcl-1 ratio surpassed the one seen in cells treated with PDT alone.

### 3.8. Specific Silencing of Noxa Inhibits Apoptosis Mediated by PDT/ABT-263

To assess whether Noxa contributed to the molecular mechanism causing the increased pro-apoptotic action of PDT in combination with ABT-263, NOXA expression was knocked down in U251 cells by specific siRNA prior to treatment with PDT in combination with ABT-263 (Figure 4d). As shown in Figure 4b,c, cells receiving a treatment with PDT in combination with ABT-263 in the presence of non-targeting siRNA displayed a significant increase in apoptotic (Annexin V-positive) cells. However, those cells silenced for Noxa showed a significantly reduced pro-apoptotic effect.

### 3.9. Combined Treatment with ABT-263 and PDT Causes Transcriptional Downregulation of Mcl-1 and Sustained ER-Stress

We next examined whether Mcl-1 or Noxa (*PMAIP1*) expression was affected by the combination therapy on the transcriptional level. We therefore performed PCR analyses to determine *Mcl-1* and *PMAIP1* mRNA levels. As shown in Figure 4e, ABT-263 treatment and PDT combined induced a significant decrease in *Mcl-1* transcripts in ULM-GBM-SC40 cells following 6 h of treatment. Regarding *PMAIP1,* while PDT alone caused an upregulation of *PMAIP1* after 6 h of treatment, both ABT-263 treatment alone and the combination therapy led to a decrease in *PMAIP1* transcripts in ULM-GBM-SC40 cells for at least 48 h (Figure 4f). Notably, the transcriptional regulation of Noxa by the combination therapy seemed to be independent of an ATF4-mediated ER-stress response. As shown in Figure 4g, PDT caused an early increase in *ATF4* expression at 6 h. Similarly, an increase in *ATF4* expression was noted at 6 h in cells that were subjected to a treatment with PDT/ABT263. However, this finding was not statistically significant. In ABT-263 treated cells, expression of *ATF4* was increased at 24 h. A sustained increase in *ATF4* after 48 h of treatment was found solely in cells subjected to the combination therapy. In line with this finding, mRNA expression of the ER-stress marker *DDIT3* (CHOP) was already increased at 6 h and still elevated after 48 h of treatment with ABT-263 plus PDT. In addition, *HSPA5* (GRP78) mRNA expression was increased in a significant manner following 48 h of treatment with PDT/ABT263.

### 3.10. Combined Treatment with ABT-263 and PDT Enhances the Stability of Noxa

**Figure 5:**
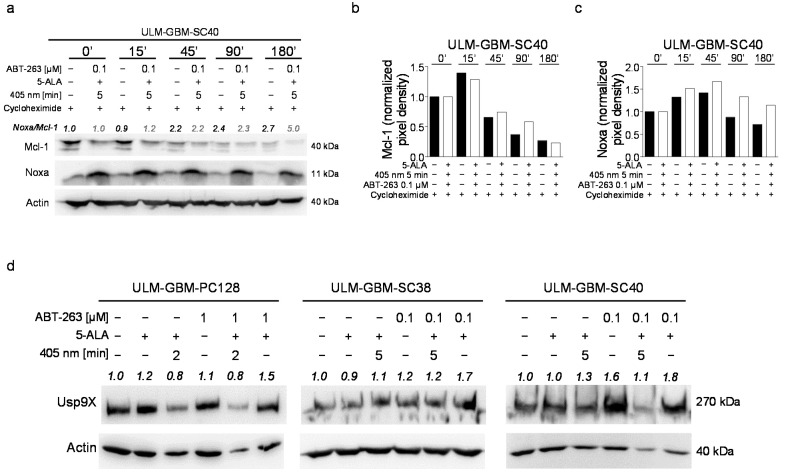
Combined treatment with PDT and ABT-263 enhances the stability of Noxa. (**a**) Cycloheximide (10 µg/mL) was added to ULM-GBM-SC40 cells after being treated with PDT/ABT-263 or solvent for 24 h. Cell lysates were harvested at defined time points and changes in protein expression of Mcl-1 and Noxa were analyzed by Western blot. Loading was controlled by Actin. Image J (NIH, Bethesda, MD; http://imagej.nih.gov/ij (accessed on 10 July 2021)) was used for densitometry. Normalization to Actin and to time 0 of the corresponding treatment group was done. (**b**,**c**) Column graphs of the Mcl-1 (**b**) or Noxa (**c**) expression of ULM-GBM-SC40 cells subjected to the same treatment as outlined for (**a**). Normalization to Actin and to time 0 of the corresponding treatment group was done. (**d**) Indicated glioblastoma cells were treated for 24 h as outlined. Cell lysates were harvested. Protein expression of Usp9X was analyzed by Western blot. Loading was controlled by Actin. Image J (NIH, Bethesda, MD; http://imagej.nih.gov/ij (accessed on 10 July 2021)) was used for densitometry. Normalization to Actin and to control was performed. The uncropped western blots are in Figure S2.

The next question we addressed was whether the combination therapy had an effect on the protein stability of Noxa or Mcl-1. ULM-GBM-SC40 cells were subjected to treatment with the combination therapy or solvent prior to addition of the translation inhibitor cycloheximide. Then, a thorough analysis of the changes in protein levels over time of Mcl-1 and Noxa was performed (Figure 5a–c). The time course of Mcl-1 protein expression did not vary among ULM-GBM-SC40 cells subjected to a simultaneous treatment with PDT and ABT263 in comparison to control (Figure 5a,b). In accordance with this finding, expression of Usp9X, a deubiquitinase that is known to stabilize Mcl-1, was not significantly reduced in cells receiving a combined treatment with PDT and ABT-263 (Figure 5d). In contrast to Mcl-1, the expression of Noxa was markedly more stable in glioblastoma cells treated with ABT-263 and PDT when compared to cells treated with solvent (Figure 5a,c).

## 4. Discussion

Photodynamic therapy relies on the basic principle of specific uptake of a photosensitive molecule or selective uptake of a precursor to a photosensitive molecule to target cells, followed by activation by light at a specific wavelength [8]. As a result of this process, biological responses such as the generation of reactive oxygen species and, ideally, selective cytocidal effects on target cells occur. 5-ALA is metabolized to the fluorescent PpIX and was shown to be safe in guiding surgical treatment of high-grade gliomas [34]. More recently, the spotlight shifted toward attempts at using the 5-ALA-based phototoxic effects of PpIX as a therapeutic means for patients with high-grade gliomas [8]. Reports from preliminary studies seem promising [35]; however, evidence from randomized controlled trials supporting a clinical benefit of PDT in glioblastoma patients is not yet available.

One important limitation to PDT as a therapeutic is the heterogenous intracellular accumulation of PpIX, especially in solid malignancies with a molecular nature as diverse as glioblastoma [36]. Bonin et al. examined the molecular characteristics of fluorescent and non-fluorescent viable tumor tissue displaying similar histological morphology [37]. Based on gene expression analyses, non-fluorescent tumor tissue was associated with a signature related to the neural subtype. In contrast, samples from fluorescent glioblastoma tissue did not clearly relate to the signature of one specific subtype but displayed molecular features shared by all subtypes. Notably, the expression of genes attributed to the PpIX biosynthetic pathway did not significantly vary among fluorescent and non-fluorescent glioblastoma tissue. As a consequence, multi-targeting strategies seem necessary to avoid therapeutic resistance to PDT. This notion is supported by past experience with targeted agents, which showed that addition of a single therapeutic measure to current first-line therapy did not succeed in altering the clinical course of glioblastoma patients in a meaningful way [38,39,40].

Other groups have already explored ways of enhancing the anti-glioblastoma activity of PDT in combinatorial approaches. One strategy focuses on increasing the accumulation of PpIX in cells to obtain a stronger phototoxic effect. Blake et al. examined whether the combination of 5-ALA and iron-chelating agents increases the collection of PpIX in different tumor cells, including U87MG glioblastoma cells [41,42]. After 3 h of incubation, combined treatment with 5-ALA and the iron chelator CP94 resulted in a significantly enhanced PpIX fluorescence and related cytotoxicity. Another group reported similar results using the inhibitory effect of gefitinib on ABCG2-mediated PpIX efflux [43]. In addition, gefitinib is an inhibitor of HER1/EGFR, which adds another potential anti-neoplastic mechanism of action to this combinatorial approach. Along this line, Fisher et al. combined a liposomal formulation of lapatinib, another HER1/EGFR inhibitor, with 5-ALA-based PDT [44]. In this study, accumulation of PpIX was significantly enhanced by additional treatment with lapatinib, and the LD_50_ of PDT was significantly reduced in the presence of lapatinib in U87 and U87vIII glioblastoma cells. In vivo, the combination treatment significantly prolonged survival in a U87 and a patient-derived glioma stem-like cell xenograft model. Notably, in U373 cells harboring either wild-type or mutated HER1/EGFR the positive effects of this combination therapy were not found or even reversed by additional treatment with lapatinib in vitro. This suggests that the therapeutic expansion of PDT by only one more measure with mechanisms different from PDT for its anti-cancer activity might be insufficient to face the vast intra-tumoral heterogeneity encountered in glioblastoma.

In terms of this study, we found that PDT at 405 nm wavelength induces apoptosis in a caspase-dependent manner. More interestingly, we observed that PDT induces a marked increase in the NOXA/Mcl-1 ratio as a possible reason for the pro-apoptotic shift following PDT. ABT-263 inhibits Bcl-2 and Bcl-xL but not Mcl-1, which opens a door to therapeutic resistance to this compound. Therefore, inhibition of Mcl-1 by increasing the expression of its natural counterpart (Noxa), which occurs in this study as a consequence of PDT, offers a vulnerability, an Achilles heel, to ABT-263 and synergistically increases its pro-apoptotic activity. While others have shown before that PDT induces a pro-apoptotic phenotype, for instance, by increasing the BAX/Bcl-2 ratio [45], this study for the first time shows that further manipulation of a PDT-induced dysbalanced apoptotic homeostasis by a pro-apoptotic small molecule inhibitor can be used to achieve synergistic pro-apoptotic effects. Furthermore, the increase in the Noxa/Mcl-1 ratio seems related to a transcriptional reduction of Mcl-1 levels and enhanced protein stability of Noxa. Notably, we observed that the protein stability of Mcl-1 was not affected by the combination therapy. These data are supported by the finding that expression of the deubiquitinase Usp9X was not significantly reduced following combined treatment with PDT and ABT-263, either. Usp9X is a well-described chaperone of Mcl-1 [46]. Therefore, downregulation of Usp9X would have favored the proteasomal degradation of Mcl-1. In addition, PDT causes early ER-stress, which is sustained by concomitant treatment with ABT-263.

Overall, our data provide proof of principle for an enhanced pro-apoptotic activity of PDT when combined with the BH-3 mimetic ABT-263. However, this study has its limitations. All experiments were performed in an in vitro setting using standardized culture conditions and, therefore, cannot take into account influences of the tumor microenvironment or immunological responses. From a translational perspective, though, the strategy proposed in this study seems feasible. PDT is clinically used, and a randomized controlled trial using interstitial PDT is on its way. ABT-263 has been clinically investigated and showed a good tolerability. The results of this study favor further pursuit of this approach by additional investigation.

## Figures and Tables

**Table 1:** Outline of the sequences of the primers.

Gene	Forward Sequence	Reverse Sequence
ATF4	TTC TCC AGC GAC AAG GCT AAG G	CTC CAA CAT CCA ATC TGT CCC G
DDIT3	GGT ATG AGG ACC TGC AAG AGG T	CTT GTG ACC TCT GCT GGT TCT G
HSPA5	CTG TCC AGG CTG GTG TGC TCT	CTT GGT AGG CAC CAC TGT GTT C
PMAIP1	CTG GAA GTC GAG TGT GCT ACT C	TGA AGG AGT CCC CTC ATG CAA G
Mcl-1	CCA AGA AAG CTG CAT CGA ACC AT	CAG CAC ATT CCT GAT GCC ACC T
18S	GTC TCC TCT GAC TTC AAC AGC G	ACC ACC CTG TTG CTG TAG CCA A

**Table 2:** Bliss analysis for indicated cells receiving the combination therapy.

Type of Cell	True Effect/Expected Effect
U251	2.51
ULM-GBM-PC38	1.13
ULM-GBM-SC38	1.11
ULM-GBM-SC40	1.12
ULM-GBM-SC128	1.53

## Data Availability

The data supporting the results reported in the article are deposited at the translational brain tumor research laboratory, Department of Neurological Surgery, Ulm University Medical Center, Albert-Einstein-Allee 23, D-89081 Ulm, Germany.

## Supplementary Materials

The following are available online at https://www.mdpi.com/article/10.3390/cancers13164123/s1, Figure S1: (a), U251 cells were subjected to solvent, 1.5 µM ABT-263 (ABT), 25 µg/mL 5-ALA combined with exposure to light with a wavelength of 405 nm for 2 min (PDT) or PDT plus ABT. After 6 h or 24 h, staining with 2′,7′-Dichlorodihydrofluorescein diacetate (H2DCFDA) and flow cytometry was performed. Histograms are shown representative for 3 independent experiments. (b), Quantitative representation of U251 cells subjected to the same treatment as outlined in b. Columns, mean; bars, SD; *n* = 3. * *p* < 0.05, *** *p* < 0.005, Figure S2: uncropped Western blots.
