# Supplementary material for: Photodynamic Therapy Combined with Bcl-2/Bcl-xL Inhibition Increases the Noxa/Mcl-1 Ratio Independent of Usp9X and Synergistically Enhances Apoptosis in Glioblastoma
Source: Cancers (Basel). 2021 Aug 17;13(16):4123. doi: 10.3390/cancers13164123 (PMC8393699; doi:10.3390/cancers13164123)
Supplement: Supplementary file 1 [file cancers-13-04123-s001.zip › cancers-1324345-Figure S1.pdf]

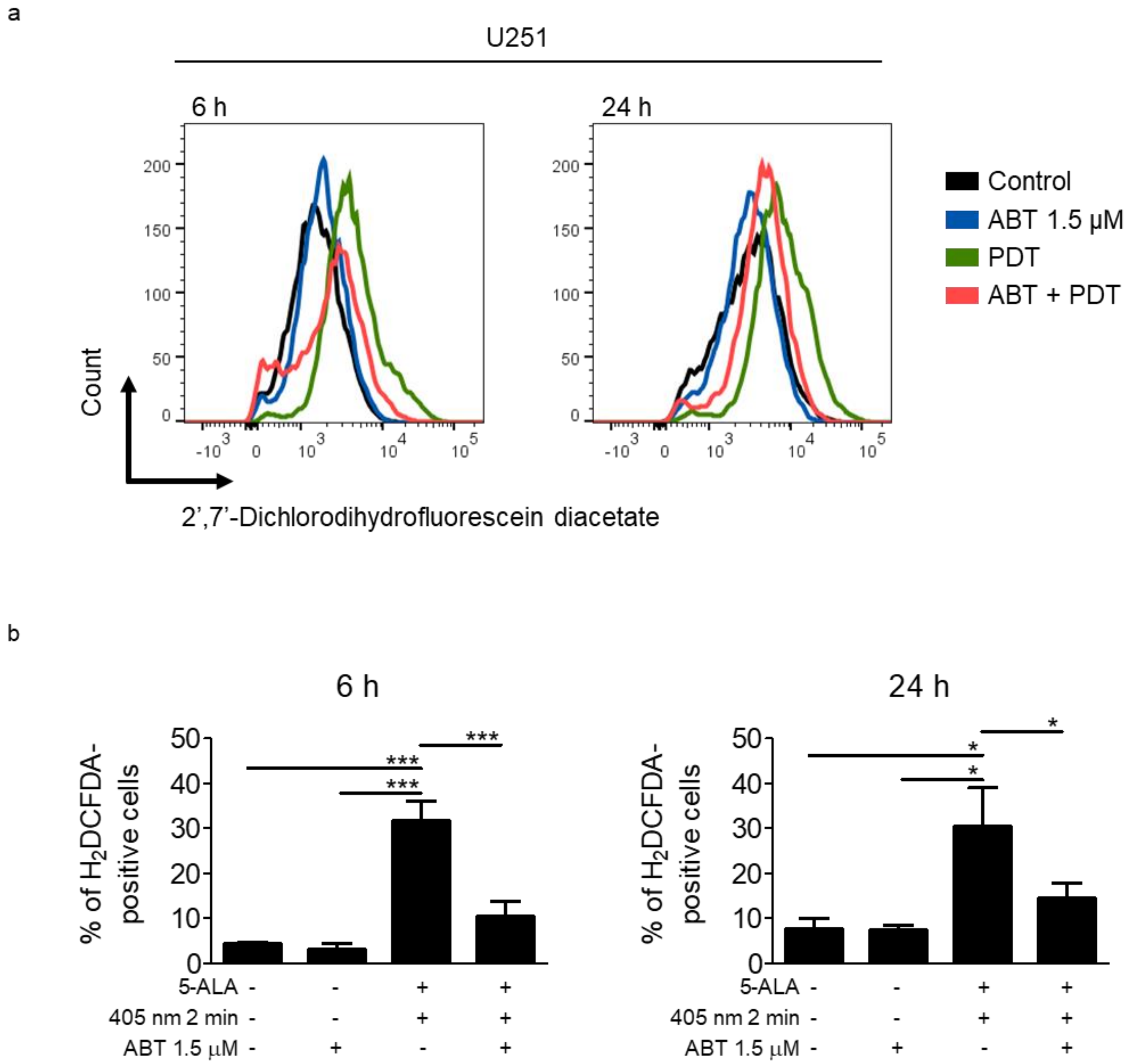

Figure S1. (a), U251 cells were subjected to solvent, 1.5 µM ABT-263 (ABT), 25 µg/ml 5-ALA combined with exposure to light with a wavelength of 405 nm for 2 min (PDT) or PDT plus ABT. After 6 h or 24 h, staining with 2',7'-Dichlorodihydrofluorescein diacetate (H_2DCFDA) and flow cytometry was performed. Representative histograms are shown representative for 3 independent experiments. (b), Quantitative representation of U251 cells subjected to the same treatment as outlined in b. Columns, mean; bars, SD; n=3. *p<0.05, ***p<0.005.
