# Supplementary figures and images for: Photodynamic Therapy Combined with Bcl-2/Bcl-xL Inhibition Increases the Noxa/Mcl-1 Ratio Independent of Usp9X and Synergistically Enhances Apoptosis in Glioblastoma
Source: Cancers (Basel). 2021 Aug 17;13(16):4123. doi: 10.3390/cancers13164123 (PMC8393699; doi:10.3390/cancers13164123)

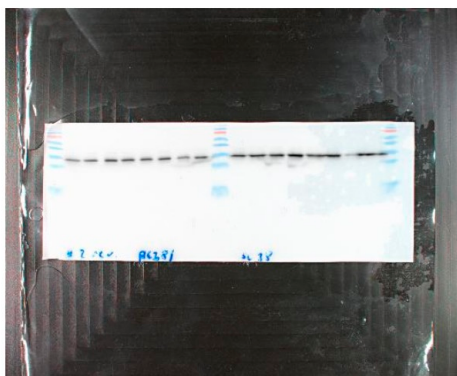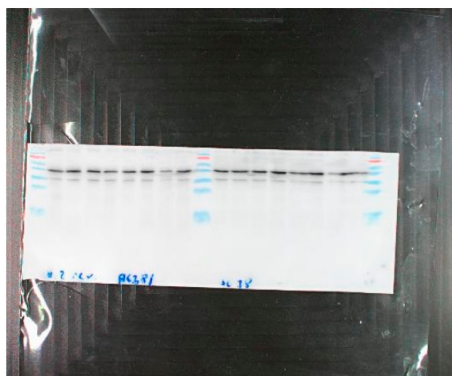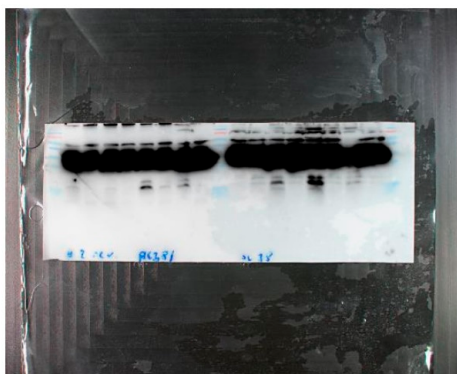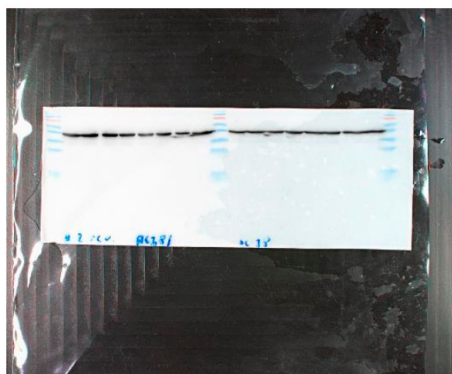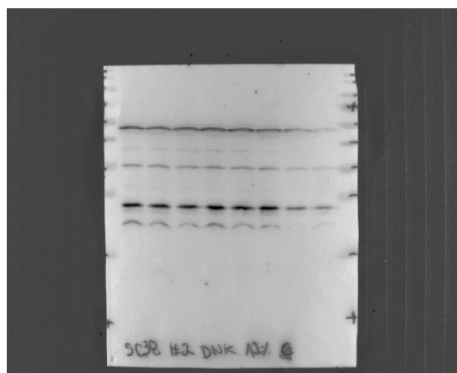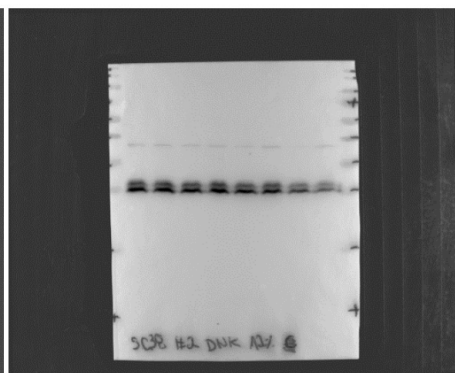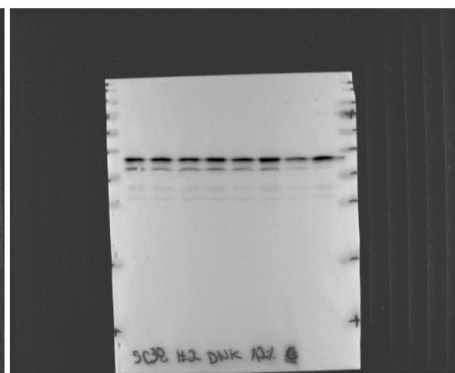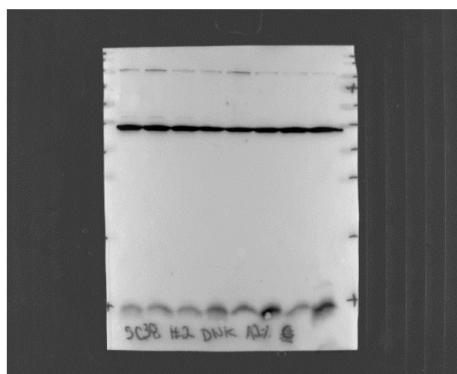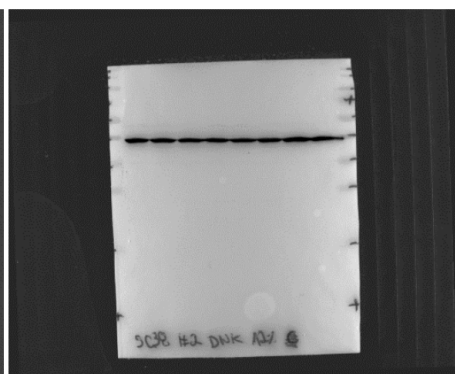

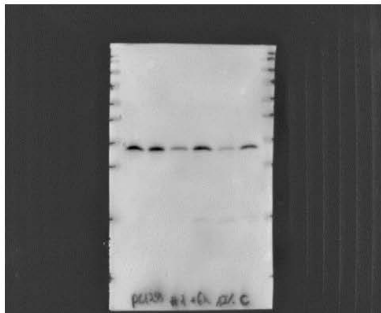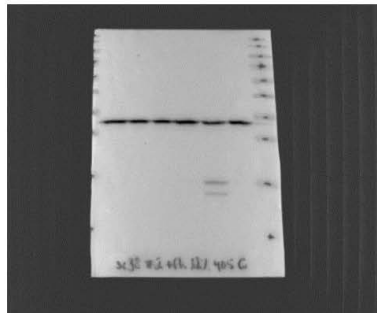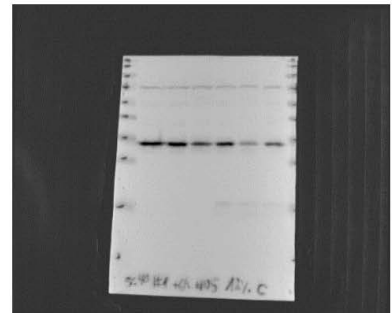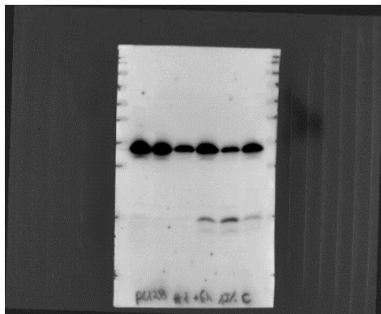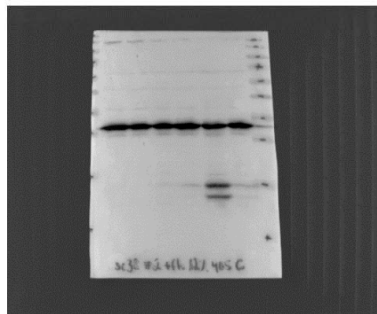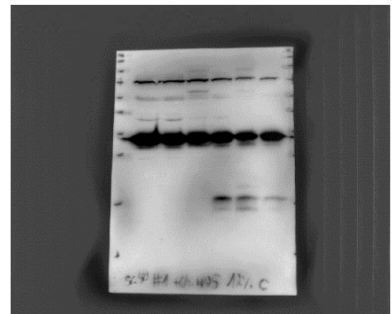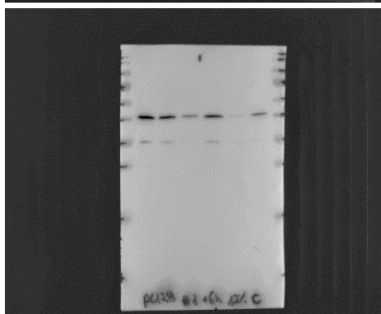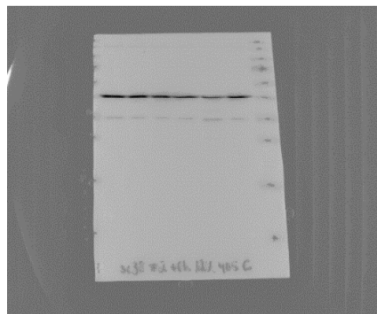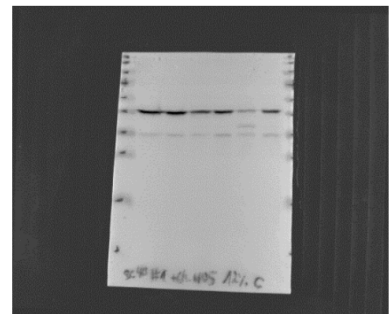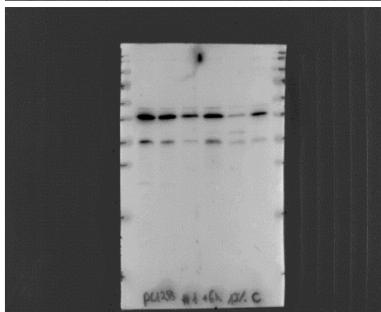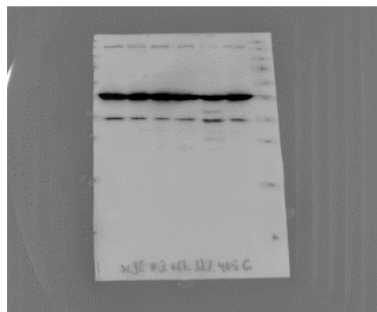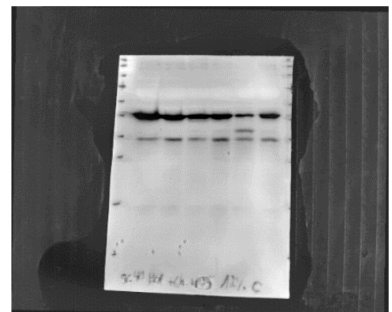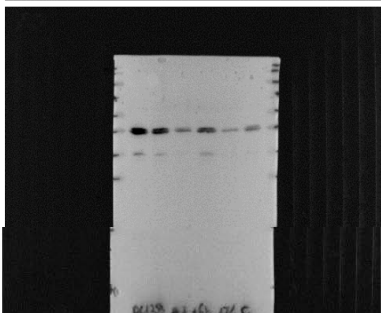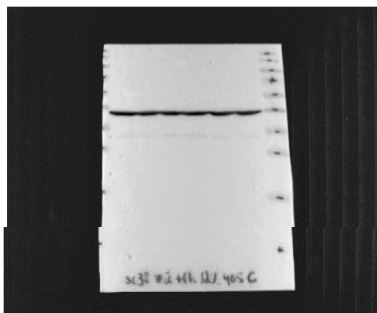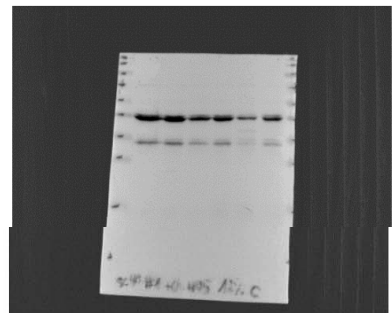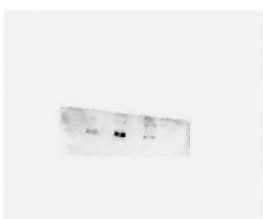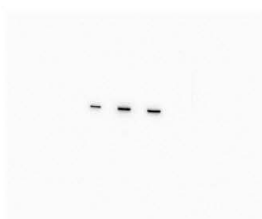

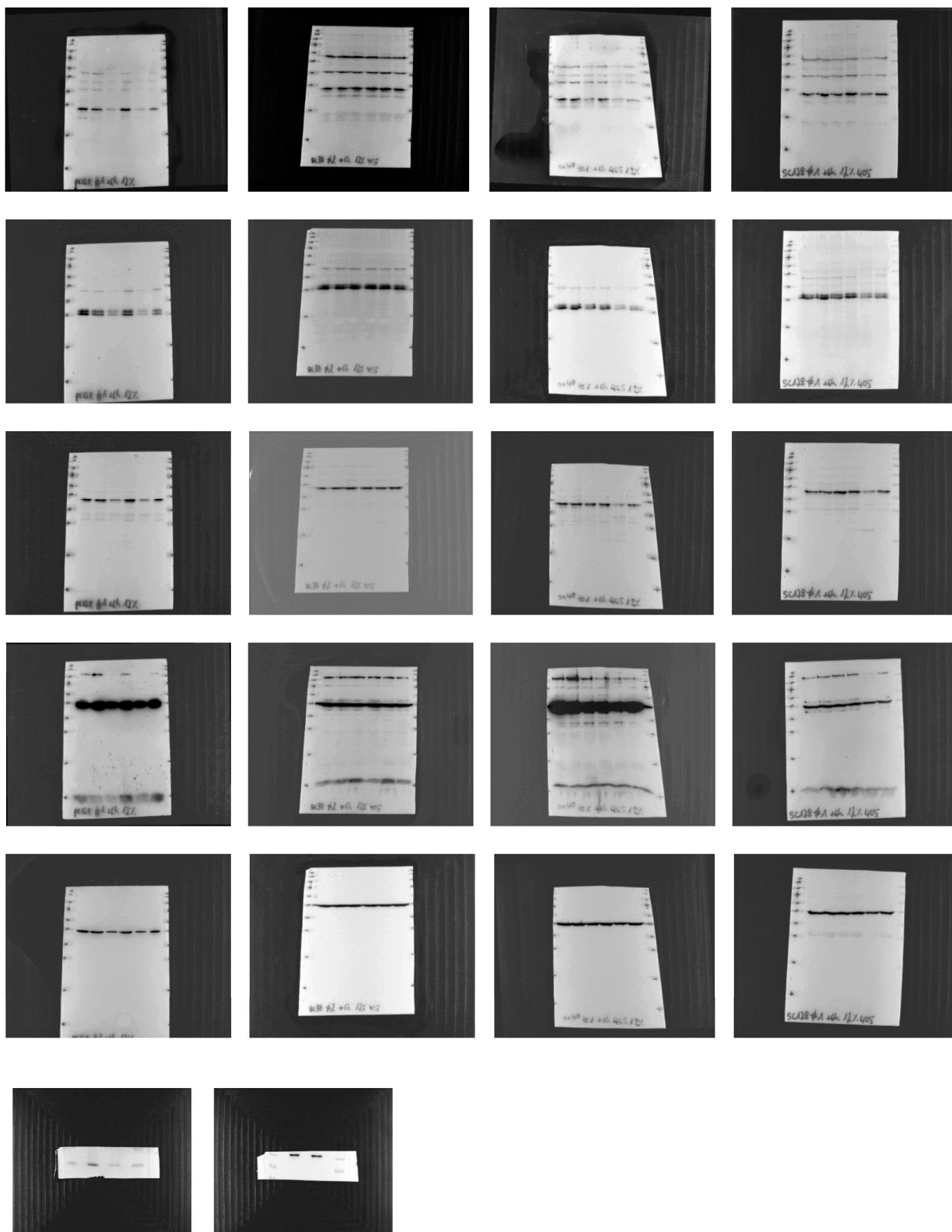

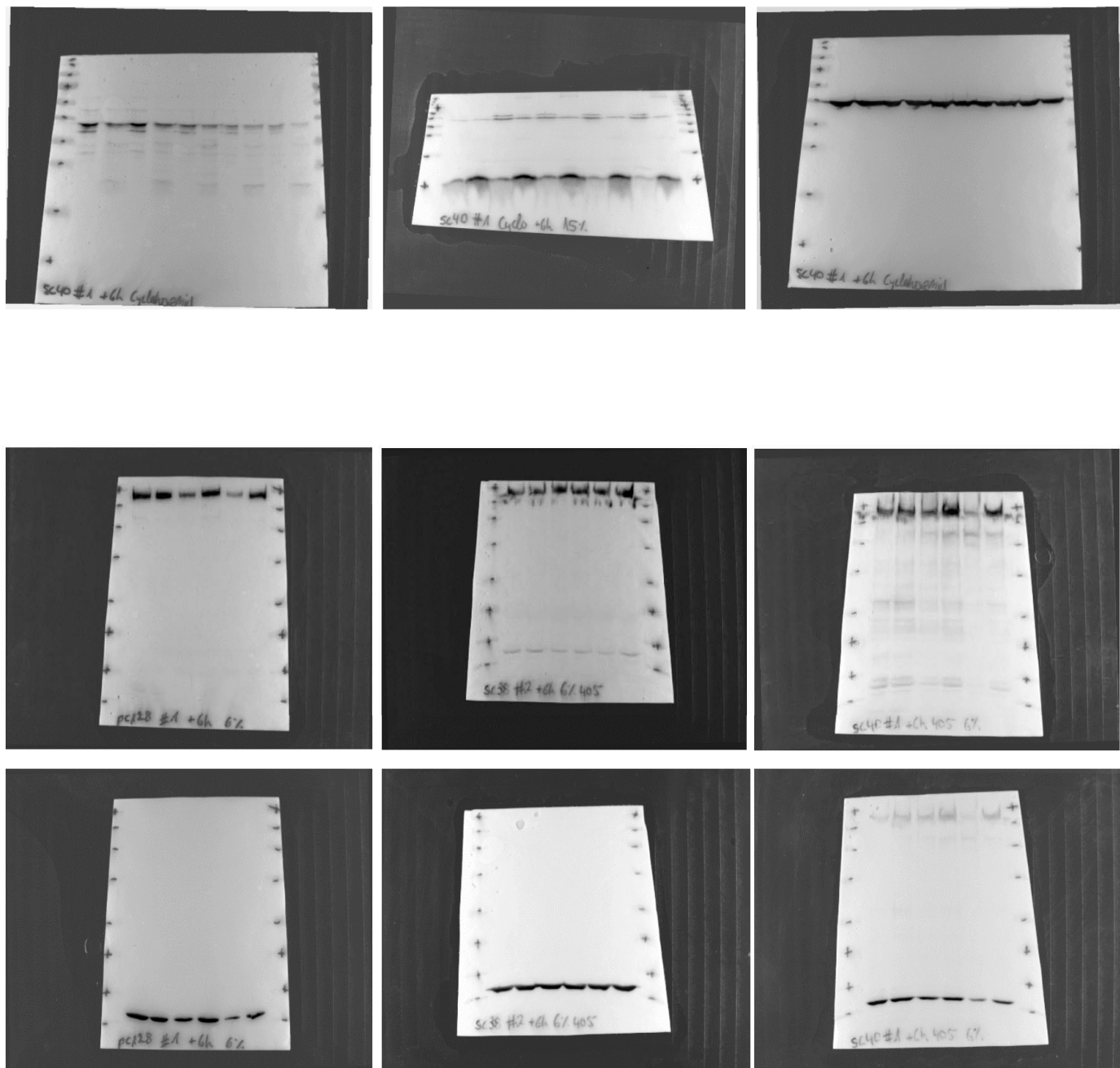

**Figure S2.** Uncropped Western Blots.

Supplement: Supplementary file 1 [file cancers-13-04123-s001.zip › cancers-1324345-Figure S2.pdf]
